# On Plugging the Vagina with Vulcanized Caoutchouc

**Published:** 1850-03

**Authors:** 


*On Plugging the Vagina with Vulcanized Caoutchouc.* By M. Diday.—M. Diday, having a case of *metrorrhagia*, in which the patient had become reduced to almost the lowest point of exhaustion, resolved to avail himself of one of Dr. Gariel's ingenious applications of vulcanized caoutchouc. The apparatus consists of a small bladder of caoutchouc, to which is attached a long tube. Rolled up so as not to exceed the little finger in size, it was passed as deeply into the vagina as possible, and kept there by the end of the finger; it was then inflated through the tube, until the small body which had been introduced almost imperceptibly, acquired a volume constituting a sphere of about 33 centimetres in diameter. The air was retained by tying the tube. No means of retaining it *in situ* were required, and the hemorrhage entirely ceasing, it was removed sixty-four hours after, as easily as introduced, by allowing the air to escape through the tube. Any one who is aware of the pain and uncertainty attending the ordinary mode of plugging the vagina, must agree with M. Diday, that this is a most useful apparatus. Its advantages consist, he adds—1. In its simplicity, and the rapidity with which it may be employed. Thus, it only weighs about half an ounce, is soft and flexible, admitting of being put in the instrument case, and is applied in a few seconds. 2. It causes no pain either during or after its application, and requires no bandage to retain it. 3. It admits before insufflation, of being moulded on the parts to be compressed, and thus can exert compression upon a cavity, however irregular in form. 4. It allows of any degree of diminution or increase of pressure to be made, according to the exigencies of the case. 5. It is impermeable to, and incorruptible by, whatever discharges it comes into contact with, and never loses its elasticity. 6. Distended only to a third or fourth of its natural extensibility, it is just as smooth, and possesses nearly as great a resisting power, as when fully distended. 7. A somewhat larger apparatus would be available for plugging the cavity of the uterus itself, in hemorrhage after delivery. Moulded on the inner surface of that organ during its state of inertia, as this became recovered from, the air would be gradually let out, and the size of the compressing vessel diminished, *pari passu* with that of the uterine cavity.

Dr. Gariel has availed himself of the remarkable properties of the vulcanized caoutchouc (its unalterability by corrosives, its preservation of elasticity at all temperatures, its great strength, and its resumption of its original size after however great extension) for the construction of a vast variety of surgical apparatus, some of which exhibit great ingenuity. Thus, there are bandages, means for making extension and counter-extension, means of exerting compression from within, as in stricture of the urethra, plugging the nares, plugging the vagina, pessaries, or from without, as in hernia, and other pads. One of the most simple of these is a portable urinal, which is of such trifling size and weight, as to cause no inconvenience or ill appearance. The penis is adapted to the orifice of this, just as the wrist is to the India-rubber band of a glove, and the material being impermeable, no smell issues. When opportunity offers, without displacing the vessel, the patient discharges the collected urine by means of a little cock attached to it.—*Ibid, from Gaz. des Hopitaux.*

